# Association Between Public Opinion and Malaysian Government Communication Strategies About the COVID-19 Crisis: Content Analysis of Image Repair Strategies in Social Media

**DOI:** 10.2196/28074

**Published:** 2021-08-04

**Authors:** Nasaai Masngut, Emma Mohamad

**Affiliations:** 1 Malaysian Nuclear Agency Bangi Malaysia; 2 Centre for Research in Media and Communication Faculty of Social Sciences and Humanities Universiti Kebangsaan Malaysia Bangi Malaysia; 3 UKM x UNICEF Communication for Development Centre in Health Faculty of Social Sciences and Humanities Universiti Kebangsaan Malaysia Bangi Malaysia

**Keywords:** COVID-19, crisis, health communication, image repair, Malaysian government, sentiment, communication, content analysis, public opinion, social media, strategy

## Abstract

**Background:**

The COVID-19 health crisis has posed an unprecedented challenge for governments worldwide to manage and communicate about the pandemic effectively, while maintaining public trust. Good leadership image in times of a health emergency is paramount to ensure public confidence in governments’ abilities to manage the crisis.

**Objective:**

The aim of this study was to identify types of image repair strategies utilized by the Malaysian government in their communication about COVID-19 in the media and analyze public responses to these messages on social media.

**Methods:**

Content analysis was employed to analyze 120 media statements and 382 comments retrieved from Facebook pages of 2 mainstream newspapers—Berita Harian and The Star. These media statements and comments were collected within a span of 6 weeks prior to and during the first implementation of Movement Control Order by the Malaysian Government. The media statements were analyzed according to Image Repair Theory to categorize strategies employed in government communications related to COVID-19 crisis. Public opinion responses were measured using modified lexicon-based sentiment analysis to categorize positive, negative, and neutral statements.

**Results:**

The Malaysian government employed all 5 Image Repair Theory strategies in their communications in both newspapers. The strategy most utilized was reducing offensiveness (75/120, 62.5%), followed by corrective action (30/120, 25.0%), evading responsibilities (10/120, 8.3%), denial (4/120, 3.3%), and mortification (1/120, 0.8%). This study also found multiple substrategies in government media statements including denial, shifting blame, provocation, defeasibility, accident, good intention, bolstering, minimization, differentiation, transcendence, attacking accuser, resolve problem, prevent recurrence, admit wrongdoing, and apologize. This study also found that 64.7% of public opinion was positive in response to media statements made by the Malaysian government and also revealed a significant positive association (*P*=.04) between image repair strategies utilized by the Malaysian government and public opinion.

**Conclusions:**

Communication in the media may assist the government in fostering positive support from the public. Suitable image repair strategies could garner positive public responses and help build trust in times of crisis.

## Introduction

### Background

A public health crisis is often a threat to health infrastructure because it can cripple an existing health care system on a larger scale. As such, a global pandemic such as the COVID-19 pandemic, could potentially be damaging to a national health system [1,2] and the stability of a country. Much of the success of a public health campaign is determined by how far the public trust their government and health institution. During the 2018 Ebola outbreak in the Democratic Republic of Congo, the federal government faced a critical challenge in disseminating relevant information, obtaining local cooperation, and conducting mass inoculation programs. This was due to a weak social system and diminished public trust. The civil unrest and domestic terrorism in the Democratic Republic of Congo made it more difficult for the government to combat the Ebola virus [3].

In Malaysia, the sudden outbreak of severe acute respiratory syndrome (SARS) in 2003 initially proved too much for the government to handle. Constant communication on SARS in the mass media led to a mild panic among the public [4]. However, the Ministry of Health played a vital role in mitigating the situation by providing constant situational reports and mobilizing a crisis management team. The Ministry also solidified their role as the main source of information to the public through daily updates on SARS and active public engagements [4]. Communication from health authorities is important to ensure that the public take necessary actions to control the spread of infectious diseases [5,6] by framing issues that are deemed critical for public’s safety [7].

While keeping the public informed remains the main goal of crisis communication, earning public trust is another matter. Gaining public trust is often achieved by using credible means [8], which can effectively improve message reception. However, social media has become an important source of information regardless of its authenticity and reliability [9]. The spread of misinformation has proved to be dangerous in times of crisis because it can easily undermine health efforts and sow the seeds of doubt among the public. Earning trust is vital to allow the government to implement necessary measures and obtain cooperation from the public.

COVID-19 was first detected in Malaysia in January 2020. Since then, the Malaysian Government through the Ministry of Health has been consistent in delivering daily updates on COVID-19 to the public. As part of their integrated effort in combatting COVID-19, the Malaysian government has activated its Crisis Preparedness and Response Center, which is a part of an overall strategy to overcome the pandemic. This center acted as a central command for early outbreak coordination and management control over the pandemic [10].

Even though the Malaysian government was generally commended for their swift action during the initial stage of local COVID-19 transmission, there were several challenges to secure public’s trust at the time. This was largely due to an unexpected political upheaval after the Prime Minister’s resignation in early March 2020. The sudden government transition itself puts Malaysia in a fragile state because of uncertainty about government stability and the capability of the newly formed government to combat COVID-19 [11]. In addition, a premature statement made by the Health Minister, suggesting that warm water might kill COVID-19 virus, was ridiculed, which did not help to foster public trust [12]. This controversial statement was later criticized by many medical practitioners who refuted the claim. In another occasion, the minister was teased for his lack of general knowledge when he accidentally claimed that 500 countries had participated in a recent World Health Organization video conference [13]. Controversial statements such as these could damage government’s reputation and jeopardize public’s trust in the government’s ability to manage the pandemic. Furthermore, several statements from the government were found to be contradictory [14], which led to escalating confusion among the public. Even though providing contradictory information in early phases of a crisis is not uncommon [15], such inconsistency may lead to further distrust among the public.

Therefore, we examined how the Malaysian government managed crisis communications on COVID-19 in the media. Specifically, this study identifies image repair strategies utilized by the Malaysian government in the media and examines how society react to these strategies by observing the directions of public sentiments through social media responses. Findings from this research could be useful in planning better crisis communication strategies.

### Measures to Mitigate COVID-19

Daily updates on COVID-19 were given by the Director General of Health, while the Minister of Defense was in charge of communicating standard operating procedures and public measures to mitigate the spread of the virus. These updates were given through separate press conferences and published in different forms of mass media, including Telegram, a freeware instant messaging app that could reach millions of people rapidly. In addition, to ensure information on COVID-19 was disseminated through proper channels, a dedicated website was set up under the purview of Ministry of Health [16]. This website is a centralized platform for daily updates on cases as well as any new developments on COVID-19, in addition to the daily press conferences. To step up their effort in curbing COVID-19, the Malaysian government enacted a Movement Control Order on March 18, 2020 for a period of 2 weeks, then extended the order by 5 weeks (for a total 7 weeks). The Movement Control Order was executed under the purview of the National Security Council under the Infectious Disease Control and Prevention Act 1988, as well as the Police Act 1967 [17]. This special measure allowed the government to enforce various movement restrictions on general population, in order to reduce virus transmission in the population.

Key economic areas and activities came to halt, along with education sector, religious houses, and sports and cultural activities. However, movement restriction exceptions were given for essential services. The implementation of Movement Control Order later transitioned to a Conditional Movement Control Order, which eased economic restriction while maintaining movement restriction. When the local case transmission showed improvement, the government introduced a Recovery Movement Control Order, which allowed gradual reopening of most key sectors. Systematic implementation of the different levels of Movement Control Order demonstrated the Malaysian government’s commitment in tackling COVID-19 nationwide. The Malaysian government also implemented MySejahtera, a contact-tracing system mobile device app. MySejahtera provided a database of COVID-19 cases in communities and charts of patients’ prior movements, to help warn the public about hotspots [18]. Some 6.2 million users downloaded the MySejahtera app by July 2020, indicating a high-level voluntary usage of the tracking app among the public [19]. The app also received an award at the Ministry of Health Innovation Day 2020 that recognized its robust features and contribution in tackling the pandemic [20]

### Image Repair Framework

This study utilized Image Repair Theory [21] to analyze government communications in the media. The theory proposed 5 main strategies and 15 substrategies of image repair. The main strategies proposed by the Image Repair Theory are denial, evasion of responsibility, reducing offensiveness, corrective action, and mortification. These strategies can be utilized independently or collectively to improve public perception. Image repair strategies are frequently used by organizations in crisis to gain favorable responses from the public. Studies [22-24] have also showed how different image repair strategies were able to help improve government’s image in crisis situations.

One study [25] suggested that a majority of the public form their perception of risk of a public health crisis from the media they consume. Various media frames may be used to deliver public health messages to help contextualize the message and urge the public to take action. Among the commonly utilized frames are risk magnitude, self-efficacy, episodic framing and economic uncertainty, which are used to communicate symptoms, likeliness to contract a disease, or protective measures the public may undertake [7]. Restoring damaged perceptions may require effective framing contexts to deliver key messages to the public. This is because the media often use sensationalism to ensure engagement from the audience [8] and this may jeopardize individual’s or organization’s image and reputation. It has also been suggested that studying the trend of public attention in the media may also help authorities determine appropriate frames to deliver key messages effectively [26]. Even though this study did not specifically investigate framing in the media, it is important to note that media messages can play a huge role in influencing public behavior [27].

While the theory [21] categorizes image repair strategies, it does not propose that one strategy is better than the other. However, it is useful to look at how these strategies are utilized and how the public responds to them in different context. For example, a study [22] found that despite different strategies used to improve Chinese government’s image during the SARS crisis, the effort was unsuccessful due to frequent contradictory messages. In another study [23], the Chinese government was successful at restoring public’s confidence using denial, bolstering and corrective action strategies in their communications. Similar strategies were also utilized by the Saudi Arabia government when faced by accusations of terrorism back in 2003 [24].

Although prior studies have recognized the importance of image repair in various crises, investigation of public sentiment on image repair strategies has been limited. Therefore, to address this knowledge gap, we explored the following research questions: (1) What are the image repair strategies employed by the Malaysian government in their communications on COVID-19 in the media? To investigate which image repair strategies utilized in the media by the Malaysian government on COVID-19, it is pertinent to analyze media statements by government officials. These statements may contain either one or more strategies outlined in Benoit’s image repair theory. (2) What is the public sentiment towards COVID-19 media statements from the Malaysian government? Government communications on COVID-19 may bring about different kinds of sentiments from the public. As the direction of sentiment may become an initial indicator of the effectiveness of image repair efforts, this study will scrutinize public’s responses toward COVID-19 media statements by the Malaysian government. (3) What is the relationship between image repair strategies and the direction of public sentiment on COVID-19 media statements by the Malaysian government? By examining the relationship between image repair strategies and the direction of public sentiment, this study aims to describe the effectiveness of the Malaysian government’s effort in communicating about COVID-19.

## Methods

### Data Collection

This study selected 2 mainstream newspapers—Berita Harian and The Star—for data collection. Statements made by the Malaysian government were retrieved via online Facebook platforms (Berita Harian Online and The Star Facebook page). These 2 newspapers have a broad influence, with 5.7 million (Berita Harian) and 1.2 million (The Star) page followers in Malaysia. Due to their prominence and influential nature, media statements from selected officials (ie, the Prime Minister, Ministers, Director General of Health and Inspector General of Police) were chosen. Additionally, responses to questions from several ministries were also included. Online comments from the public on statements made by these government officials were also collected to answer the second research question.

### Sample Period

Key official media statements published in Berita Harian Online and The Star Facebook page pertaining to COVID-19 from March 4, 2020 (2 weeks before the implementation of Movement Control Order) to April 30, 2020 (6 weeks into the implementation of the Movement Control Order) were collected. Online comments from the statements were also extracted from the same period.

### Sample Size and Sample Data Collection

To investigate the first research question, 120 media statements were taken as a sample. Subsequently, we estimated [28] a sample size of 382 out of 59,941 online comments to examine the direction of public sentiment; this sample has a confidence level of 95% and margin of error of 5%. sampling with stratification (50% from Berita Harian Online and 50% from The Star Facebook page), by selecting top comments labeled as “most relevant” by Facebook algorithm from each media statement, was used. The algorithm sorted and ranked comments based on the highest quality of comments that are relevant to users [29]. Only comments with Malaysian names were taken as sample [30]. These comments were then included for content analysis based on image repair framework.

### Content Analysis and Coding Scheme

Content analysis was employed to identify image repair strategies utilized in the 120 online media statements by the Malaysian government and 382 comments from the public. Coding scheme for research question 1 was adapted from a previous study [31], in which 5 main strategies and 15 substrategies were used. The strategies are denial, evading responsibility, reducing offensiveness, corrective action, and mortification. The substrategies were simple denial, shifting blame, provocation, defeasibility, accident, good intention, bolstering, minimization, differentiation, transcendence, attacking accuser, fixing problem, preventing recurrence, admitting fault, and apologizing.

To answer research question 2, this study categorized sentiments by employing Valence Aware Dictionary for Sentiment Reasoning (VADER) text analysis technique [32]. Through this particular sentiment analysis method, public comments were categorized into positive, negative or neutral. Despite the language limitation of the VADER technique, which only catered to English language [33], this study was able to identify and replicate its algorithm to the Malay language for when analyzing comments in Bahasa Malaysia. To correspond with VADER analytical algorithm, 4 main principles were applied toward analyzing social media text in online comments: punctuation, capitalization, intensifiers, and conjunction [33]. Results obtained from research question 1 and research question 2 were then to measure relationships outlined in research question 3.

### Validity and Intercoder Reliability

Validity for coding instrument in content analysis can be described as face validity, construct validity, content validity, and criteria validity [34]. The coding instrument for the study has been face validated by a researcher based at the *Universiti Kebangsaan Malaysia*.

Two coders analyzed the media statements and public comments. To ensure intercoder reliability, both coders were trained using a coding scheme. Intercoder reliability [35] was calculated as *R* = 2*M* / (*N1* + *N2*), where *M* is the total mutually agreed coding result and *N1* and *N2* are numbers of decisions made by coder 1 and coder 2. Each coder analyzed 10 media statements that were not part of the sample (*R*=0.74) and 10 public comments (*R*=0.74). An *R* value equal or more than 0.70 is accepted as reliable [36].

### Statistical Analysis

A statistical analysis was performed using SPSS software (version 22; IBM Corp). For descriptive analysis, frequency tables on media source, publication phase, statement source and image repair strategies (including substrategies) were created to address research question 1. This analysis revealed highest frequency of image repair strategies and substrategies utilized by the Malaysian government. A frequency table was also generated to categorize and reveal direction of public sentiments (positive, negative or neutral) to answer research question 2. Chi-square tests were used to determine the association between COVID-19 image repair strategies by the Malaysian government and the direction of public sentiment from online comments to answer research question 3.

To measure the effect size for cross-tabulated data, Pallant [37] has suggested that for a table larger than 2 by 2 Cramer *V*, which takes into account degrees of freedom, with *V*<0.07 small, *V*=0.08-0.21 medium, and *V*>0.35 large suggested.

## Results

The *reducing offensiveness* strategy was the most utilized strategy in COVID-19 media statements (75/120, 62.5%). It was used more than the *corrective action* strategy, which was the second-most used (30/120, 25.0%) (Table 1).

For image repair substrategies, a total of 454 statements were coded (Multimedia Appendix 1). The *bolstering positive quality* substrategy (reducing offensiveness strategy) appeared the most in the media statements (108/454, 23.7%), while the *fixing problem* substrategy (under corrective action strategy) came in second (104/454, 22.9%). Other frequently occurring substrategies were preventing recurrence (102/454, 22.4%) and minimization (38/454, 5.3%).

**Table 1:** Image repair strategy frequency.

Image repair strategy	Instances, n (%)
Denial	10 (8.3)
Evade responsibility	4 (3.3)
Reduce offensiveness	75 (62.5)
Corrective action	30 (25.0)
Mortification	1 (0.8)
Total	120 (100.0)

Findings (Multimedia Appendix 2) also revealed that most media statements were made by the Director General of Health (24/120, 20.0%), followed closely by the Prime Minister and Senior Minister of Defense, (both 22/120, 18.3%). In terms of media sources, Berita Harian published more media statements from the Government on COVID-19 (87/120, 72.5%) than The Star (33/120, 27.5%). This study also observed that most media statements were published during the Movement Control Order (105/120, 87.5%) as opposed to before the Movement Control Order (15/120, 12.5%) (Multimedia Appendix 3).

A sample of Facebook comments (382/59,941) from the 120 media statements showed more positive (247/382, 64.7%), than negative (86/382, 22.5%) or neutral (49/382, 12.8%) sentiments. Berita Harian had slightly more positive sentiments (134/382, 70.9%) than The Star (112/382, 58.3%). Positive sentiments were higher in both media; however, there were more negative sentiments (Berita Harian: 34/382, 18.0%; The Star: 52/382, 27.1%) than neutral sentiments (Berita Harian: 22/382, 11.1%; The Star: 27/382, 14.6%) recorded.

The *denial* strategy received the most positive sentiments (33/47, 70.2%) (Table 2), while the *corrective action* strategy came second (42/60, 70.0%). The *reducing offensiveness* strategy, which was most utilized in Government statements, was the third highest in receiving positive sentiments (169/264, 64.0%). There was a significant association between image repair strategies of the Malaysian government COVID-19 media statements and the direction of public sentiment with a small effect (n=382, χ_8_^2^= 0.146, *P*=0.039 Cramer *V*=0.039).

**Table 2:** Direction of sentiment.

Direction of sentiment	Strategy, n (%)				
	Denial (n=47)	Evade responsibility (n=10)	Reduce offensiveness (n=264)	Corrective action (n=60)	Mortification (n=1)
Positive	33 (70.2)	2 (20.0)	169 (64.0)	42 (70.0)	1 (100.0)
Negative	6 (12.8)	4 (40.0)	66 (25.0)	10 (16.7)	0 (0)
Neutral	8 (17.0)	4 (40.0)	29 (11.0)	8 (13.3)	0 (0)

## Discussion

### Principal Results

A range of image repair strategies were employed by the Malaysian government in their COVID-19 media statements and could be categorized according to the 5 strategies suggested by Image Repair Theory [21]. In particular, the *reduce offensiveness* strategy was the most utilized strategy in communicating COVID-19 in the media, and the majority of these media statements employed the *bolstering positive quality* substrategy. There was also a significant association (*P*=.04) between image repair strategies and the direction of public sentiment. Although the *reducing offensiveness* strategy was most utilized, results showed that the *denial* strategy received the highest positive sentiments, which was followed by the *corrective action* strategy, and then the *reducing offensiveness* strategy. Nonetheless, overall sentiment towards government’s messaging were positive.

Although the image repair theory does not stipulate crisis management strategies employed by the Malaysian government, the theory provided a framework to analyze their statements in the media [38]. An understanding of how a crisis is framed by the media helps to identify strategies that might work in future crisis communications.

This study revealed that the strategy utilized most by the Malaysian government in their COVID-19 media statements was reducing offensiveness. An example of reducing offensiveness was a statement by the Health Minister explaining the need to minimize visiting hours at the hospitals as a proactive measure to reduce the risk of COVID-19 infection. A previous study [39] revealed a similar result; reducing offensiveness in the wake of Russian tourism ban to Egypt due to safety and security concern was proven to be effective and has resulted in Russian flight resuming its operations to Cairo in April 2018 after a 2-year ban.

### Implications, Limitations, and Future Work

This study contributes to current literature on COVID-19 in Malaysia [40,41] as well as understanding COVID-19 crisis communications by the Malaysian government based on the framework suggested by Benoit [21]. It adds to the body of knowledge on image repair strategies and public opinion, which may be useful in a health crisis with this global magnitude. Government efforts to mitigate the spread of COVID-19, such as enacting the Movement Control Order, have had a big impact on population, such as closure of school and non-essential businesses. Therefore, communication of these types of orders must be done correctly to not instigate fear and panic among the public [42]. Negative sentiment from the public could hamper government efforts and cause distrust in the health care delivery system.

A prior study found that, in Malaysia, television and internet news portals are primarily used to access information on COVID-19 [40] and suggested that health authorities should pay considerable attention to the use of appropriate media channels and sources to allow for more effective dissemination of critical information to the public. By identifying too which image repair strategies the Malaysian public responded well, the findings of our study provides insight into information framing that can receive positive responses from the public. We suggest using the denial, corrective action, and reducing offensiveness in television and internet news portals to communicate about crises.

The government should evaluate strengths and limitations of a country in addressing a health crisis [43]. A previous study [44] highlighted several challenges in communicating in a crisis including misinformation, lack of guidance, and information leakage. It has also been suggested that social media caused more confusion, rather than consolidating public effort against the pandemic [45]. Therefore, information in the media must focus on improving trust, building social solidarity, and reducing chaos, while educating the public on prevention measures and reducing burden on the health system. With suitable and effective image repair strategies, the government could minimize public uncertainty and mitigate the spread of false information.

One of the limitations of this study pertains to the sampling period. As the media statements and comments were captured in a specific time span, the results obtained are only applicable and true to the specific time range. Additionally, the study only took public opinions from Facebook comments into account. Results may have differed if the study extrapolated samples in other social media such as Twitter or Instagram. In addition, the consistent appearance of well-liked individuals such as the Director General of Health, may have contributed to the overall positive responses. The Director General of Health was considered a national hero and an exemplary leader in crisis, and his appearances in front of the camera to deliver daily updates on COVID-19 in a calm and composed manner has earned him the image of a rationale leader, providing assurance and tranquility to the public [46]. An investigation into the roles of frequent media appearances and leadership figures in times of crisis, as well as its influences toward public acceptance should be explored in future studies.

### Conclusions

This study provided comprehensive insight into image repair strategies in the media by the Malaysian government and how members of the public reacted in response to these strategies. The findings of this study could be useful to advise future crisis communication planning, particularly in a health crisis.

## Appendix

Multimedia Appendix 1 Image repair substrategy frequency distribution.

Multimedia Appendix 2 Source frequency distribution.

Multimedia Appendix 3 Publication phase distribution.

## Abbreviations

SARS: severe acute respiratory syndrome
VADER: Valence Aware Dictionary for Sentiment Reasoning
