# Supplementary material for: Association Between Public Opinion and Malaysian Government Communication Strategies About the COVID-19 Crisis: Content Analysis of Image Repair Strategies in Social Media
Source: J Med Internet Res. 2021 Aug 4;23(8):e28074. doi: 10.2196/28074 (PMC8341088; doi:10.2196/28074)
Supplement: Multimedia Appendix 1 [file jmir_v23i8e28074_app1.docx]

**Appendix 1**. Image repair sub-strategy frequency distribution

| **Image repair sub-strategy** | **N (%)** |
| --- | --- |
| Simple denial | 15 (3.3%) |
| Shift blame | 4 (0.88%) |
| Accidents | 3 (0.66% |
| Good intention | 27 (5.94%) |
| Provocation | 2 (0.44%) |
| Defeasibility | 11 (2.42%) |
| Transcendence | 12 (2.64%) |
| Attack accuser | 13 (2.86%) |
| Minimization | 38 (8.37%) |
| Differentiation | 8 (1.76%) |
| Bolstering | 108 (23.8%) |
| Fix problem | 104 (22.9%) |
| Prevent recurrence | 102 (22.4%) |
| Apologize | 7 (1.54%) |
| Total | 454 (100%) |
