# Supplementary material for: Association Between Public Opinion and Malaysian Government Communication Strategies About the COVID-19 Crisis: Content Analysis of Image Repair Strategies in Social Media
Source: J Med Internet Res. 2021 Aug 4;23(8):e28074. doi: 10.2196/28074 (PMC8341088; doi:10.2196/28074)
Supplement: Multimedia Appendix 2 [file jmir_v23i8e28074_app2.docx]

**Appendix 2.** Source frequency distribution

| **Statement source** | **N (%)** |
| --- | --- |
| Prime Minister | 22 (18.3%) |
| Senior Minister (Defense) | 22 (18.3%) |
| Health Director General | 24 (20.0%) |
| Inspector General | 6 (5.0%) |
| Health Minister | 5 (4.2%) |
| Finance Minister | 2 (1.6%) |
| Multimedia & Communication Minister | 1 (0.8%) |
| Foreign Minister | 3 (2.5%) |
| Science, Technology & Innovation Minister | 1 (0.8%) |
| Education Minister | 1 (0.8%) |
| Higher Education Minister | 1 (0.8%) |
| Human Resource Minister | 1 (0.8%) |
| Federal Territories Minister | 2 (1.6%) |
| Transport Minister | 2 (1.6%) |
| Senior Minister (Work) | 1 (0.8%) |
| Agro Entrepreneurship Minister | 1 (0.8%) |
| Ministries representatives | 12 (9.6%) |
| Others | 13 (10.4%) |
| Total | 120 (100%) |
