# Supplementary material for: Association Between Public Opinion and Malaysian Government Communication Strategies About the COVID-19 Crisis: Content Analysis of Image Repair Strategies in Social Media
Source: J Med Internet Res. 2021 Aug 4;23(8):e28074. doi: 10.2196/28074 (PMC8341088; doi:10.2196/28074)
Supplement: Multimedia Appendix 3 [file jmir_v23i8e28074_app3.docx]

**Appendix 3.** Publication phase distribution

| **Phase** | **N (%)** |
| --- | --- |
| Pre-MCO | 15 (12.5%) |
| MCO | 105 (87.5%) |
| Total | 120 (100%) |
